# 4-{2-[5-(4-Chloro­phen­yl)-1-(4-fluoro­phen­yl)-1*H*-pyrazol-3-yl]thia­zol-4-yl}benzonitrile

**DOI:** 10.1107/S1600536810031405

**Published:** 2010-08-11

**Authors:** Tara Shahani, Hoong-Kun Fun, R. Venkat Ragavan, V. Vijayakumar, S. Sarveswari

**Affiliations:** a X-ray Crystallography Unit, School of Physics, Universiti Sains Malaysia, 11800 USM, Penang, Malaysia; b Organic Chemistry Division, School of Advanced Sciences, VIT University, Vellore 632 014, India

## Abstract

The asymmetric unit of the title compound, C_25_H_14_ClFN_4_S, contains two independent mol­ecules (*A* and *B*). Each mol­ecule consists of five rings, namely chloro­phenyl, fluoro­phenyl, 1*H*-pyrazole, thia­zole and benzonitrile. In mol­ecule *A*, the 1*H*-pyrazole ring makes dihedral angles of 52.54 (8), 35.96 (8) and 15.43 (8)° with respect to the attached chloro­phenyl, fluoro­phenyl and thia­zole rings. The corresponding values in mol­ecule *B* are 51.65 (8), 37.26 (8) and 8.32 (8)°. In the crystal, mol­ecules are linked into dimers by C—H⋯N hydrogen bonds, generating *R*_2_^2^(10) ring motifs. These dimers are further linked into two-dimensional arrays parallel to the *ab* plane *via* inter­molecular weak C—H⋯N and C—H⋯F hydrogen bonds. The crystal structure is further stabilized by weak π-π inter­actions [with centroid–centroid distances of 3.4303 (9) and 3.6826 (9) Å] and weak C—H⋯π inter­actions.

## Related literature

For background and the microbial activity of pyrazole derivatives, see: Ragavan *et al.* (2009 ▶, 2010 ▶). For related structures, see: Shahani *et al.* (2009 ▶, 2010*a* ▶,*b* ▶). For hydrogen-bond motifs, see: Bernstein *et al.* (1995 ▶). For standard bond-length data, see: Allen *et al.* (1987 ▶). For the stability of the temperature controller used in the data collection, see: Cosier & Glazer (1986 ▶).
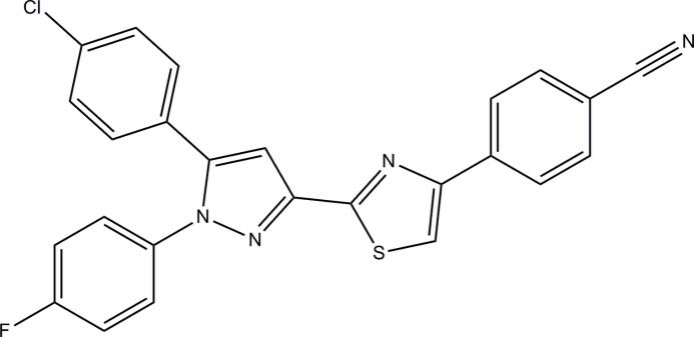


## Experimental

### 

#### Crystal data

C_25_H_14_ClFN_4_S
*M* *_r_* = 456.91
Triclinic, 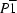

*a* = 10.1412 (9) Å
*b* = 15.0496 (14) Å
*c* = 15.8890 (14) Å
α = 105.518 (2)°
β = 107.869 (2)°
γ = 99.253 (2)°
*V* = 2144.5 (3) Å^3^
*Z* = 4
Mo *K*α radiation
μ = 0.31 mm^−1^
*T* = 100 K
0.32 × 0.26 × 0.08 mm

#### Data collection

Bruker APEXII DUO CCD area-detector diffractometer
Absorption correction: multi-scan (*SADABS*; Bruker, 2009 ▶) *T*_min_ = 0.909, *T*_max_ = 0.977
44680 measured reflections
12570 independent reflections
9300 reflections with *I* > 2σ(*I*)
*R*_int_ = 0.047

#### Refinement

*R*[*F* ^2^ > 2σ(*F* ^2^)] = 0.042
*wR*(*F* ^2^) = 0.107
*S* = 1.01
12570 reflections
577 parameters
H-atom parameters constrained
Δρ_max_ = 0.45 e Å^−3^
Δρ_min_ = −0.50 e Å^−3^

### 

Data collection: *APEX2* (Bruker, 2009 ▶); cell refinement: *SAINT* (Bruker, 2009 ▶); data reduction: *SAINT*; program(s) used to solve structure: *SHELXTL* (Sheldrick, 2008 ▶); program(s) used to refine structure: *SHELXTL*; molecular graphics: *SHELXTL*; software used to prepare material for publication: *SHELXTL* and *PLATON* (Spek, 2009 ▶).

## Supplementary Material

Crystal structure: contains datablocks global, I. DOI: 10.1107/S1600536810031405/lh5111sup1.cif

Structure factors: contains datablocks I. DOI: 10.1107/S1600536810031405/lh5111Isup2.hkl

Additional supplementary materials:  crystallographic information; 3D view; checkCIF report

## Figures and Tables

**Table 1:** Hydrogen-bond geometry (Å, °) *Cg*1 and *Cg*2 are the centroids of the C1*A*–C6*A* and C1*B*–C6*B* rings, respectively.

*D*—H⋯*A*	*D*—H	H⋯*A*	*D*⋯*A*	*D*—H⋯*A*
C5*A*—H5*AA*⋯F1*A*^i^	0.93	2.39	3.149 (2)	138
C8*B*—H8*BA*⋯F1*B*^i^	0.93	2.42	3.283 (2)	154
C17*B*—H17*A*⋯N4*A*^ii^	0.93	2.54	3.419 (2)	159
C17*A*—H17*B*⋯N4*B*^ii^	0.93	2.58	3.453 (2)	156
C25*B*—H25*A*⋯N2*A*^iii^	0.93	2.53	3.457 (2)	175
C24*A*—H24*B*⋯*Cg*1^iv^	0.93	2.96	3.7811 (18)	148
C21*B*—H21*A*⋯*Cg*2^v^	0.93	2.97	3.6423 (19)	131

Symmetry codes: (i) 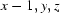; (ii) 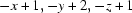; (iii) 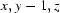; (iv) 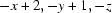; (v) 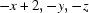.

**Fig. 1.**
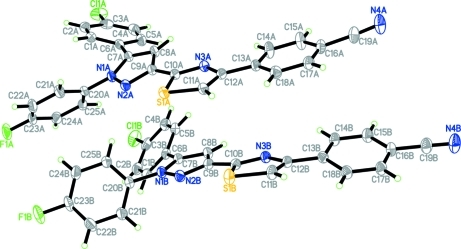
The molecular structure of the title compound, showing 50% probability displacement ellipsoids and the atom numbering scheme.

**Fig. 2.**
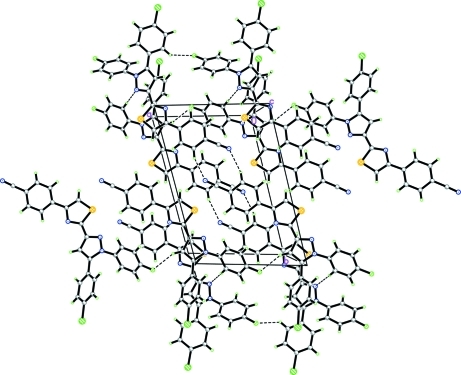
The crystal packing of the title compound, showing a two-dimensional array parallel to the ab plane. Intermolecular hydrogen bonds are shown as dashed lines.

## supplementary crystallographic information

### Comment

Antibacterial and antifungal activities of the azoles are most widely studied and some of them are used clinically as anti-microbial agents. In particular pyrazole derivatives are extensively studied and used as antimicrobial agents. However, azole-resistant strains led to the development of new antimicrobial compounds. Pyrazole is an important class of heterocyclic compounds and many pyrazole derivatives are reported to have a broad spectrum of biological activities, such as anti-inflammatory, antifungal, herbicidal, anti-tumour, cytotoxic and antiviral activities. Pyrazole derivatives also act as antiangiogenic agents, A3 adenosine receptor antagonists, neuropeptide YY5 receptor antagonists as well as kinase inhibitor for treatment of type 2 diabetes, hyperlipidemia, obesity, and thrombopiotinmimetics. Recently urea derivatives of pyrazoles have been reported as potent inhibitors of p38 kinase. Since the high electronegativity of halogens (particularly chlorine and fluorine) in the aromatic part of the drug molecules play an important role in enhancing their biological activity, we are interested to have 4-fluoro or 4-chloro substitution in the aryls of 1,5-diaryl pyrazoles. As part of our ongoing research aiming on the synthesis of new antimicrobial compounds, we have reported the synthesis of novel pyrazole derivatives and their microbial activities (Ragavan *et al.*, 2009; 2010).

The asymmetric unit of the title compound (Fig. 1) contains two molecules (*A* and *B*) with similar geometries. Each molecule consists of five rings, namely chlorophenyl (C1–C6/Cl1), fluorophenyl (C20–C25/F1), 1*H*-pyrazole (N1/N2/C7–C9), thiazole (N3/S1/C10–C12) and benzonitrile (C13–C19/N4) rings. In molecule *A*, the 1*H*-pyrazole ring is inclined at angles of 52.54 (8) and 35.96 (8)° and 15.43 (8)° with respect to the chlorophenyl, fluorophenyl and thiazole rings attached to it. The corresponding values in molecule *B* are 51.65 (8), 37.26 (8) and 8.32 (8)°. The bond lengths (Allen *et al.*, 1987), and angles are within normal ranges and comparable to the closely related structures (Shahani *et al.*,2009; 2010*a*,*b*).

In the crystal packing (Fig. 2), intermolecular C17B—H17A···N4A and C17A—H17B···N4B hydrogen bonds (Table 1) link the neighbouring molecules into dimers, generating *R*^2^_2_(10) ring motifs (Bernstein *et al.*, 1995). These dimers are further linked into two-dimensional arrays parallel to the *ab* plane by intermolecular C5A—H5AA···F1A, C8B—H8BA···F1B and C25B—H25A···N2A hydrogen bonds (Table 1). Weak π···π interactions are observed [*Cg*1···*Cg*1^vi^ = 3.4303 (9) Å, symmetry code vi = 2 - *x*, 2 - *y*, 1 - *z*], [*Cg*1···*Cg*2^vii^ = 3.6826 (9) Å, symmetry code vii = *x*, 1 + *y*, *z*] where *Cg*1 is the centroid of the thiazole ring (S1A/N3A/C10A—C12A) and *Cg*2 is the centroid of the 1*H*-pyrazole ring (N1B/N2B/C7B–C9B). The crystal structure is further stabilized by C—H···π interactions (Table 1), involving the C1A–C6A (centroid *Cg*3) and C1B–C6B rings (centroid *Cg*4).

### Experimental

The compound has been synthesized by adopting the procedure available in the literature and purified by crystallization in ethanol (Ragavan *et al.*, 2009; 2010). Yellow solid, 76% yield, *mp*: 479.9–480.8 k.

### Refinement

H atoms were positioned geometrically [C–H = 0.9300 Å] and refined using a riding model, with *U*_iso_(H) = 1.2 *U*_eq_(C).

### Crystal data

**Table:**

C_25_H_14_ClFN_4_S	*Z* = 4
*M**_r_* = 456.91	*F*(000) = 936
Triclinic, *P*1	*D*_x_ = 1.415 Mg m^−^^3^
Hall symbol: -P 1	Mo *K*α radiation, λ = 0.71073 Å
*a* = 10.1412 (9) Å	Cell parameters from 8583 reflections
*b* = 15.0496 (14) Å	θ = 2.7–30.0°
*c* = 15.8890 (14) Å	µ = 0.31 mm^−^^1^
α = 105.518 (2)°	*T* = 100 K
β = 107.869 (2)°	Plate, yellow
γ = 99.253 (2)°	0.32 × 0.26 × 0.08 mm
*V* = 2144.5 (3) Å^3^	

### Data collection

**Table:**

Bruker APEXII DUO CCD area-detector diffractometer	12570 independent reflections
Radiation source: fine-focus sealed tube	9300 reflections with *I* > 2σ(*I*)
graphite	*R*_int_ = 0.047
φ and ω scans	θ_max_ = 30.2°, θ_min_ = 1.7°
Absorption correction: multi-scan (*SADABS*; Bruker, 2009)	*h* = −14→14
*T*_min_ = 0.909, *T*_max_ = 0.977	*k* = −21→21
44680 measured reflections	*l* = −22→22

### Refinement

**Table:**

Refinement on *F*^2^	Primary atom site location: structure-invariant direct methods
Least-squares matrix: full	Secondary atom site location: difference Fourier map
*R*[*F*^2^ > 2σ(*F*^2^)] = 0.042	Hydrogen site location: inferred from neighbouring sites
*wR*(*F*^2^) = 0.107	H-atom parameters constrained
*S* = 1.01	*w* = 1/[σ^2^(*F*_o_^2^) + (0.044*P*)^2^ + 0.5673*P*] where *P* = (*F*_o_^2^ + 2*F*_c_^2^)/3
12570 reflections	(Δ/σ)_max_ < 0.001
577 parameters	Δρ_max_ = 0.45 e Å^−^^3^
0 restraints	Δρ_min_ = −0.50 e Å^−^^3^

### Special details

**Table:**

Experimental. The crystal was placed in the cold stream of an Oxford Cyrosystems Cobra open-flow nitrogen cryostat (Cosier & Glazer, 1986) operating at 100.0 (1) K.
Geometry. All e.s.d.'s (except the e.s.d. in the dihedral angle between two l.s. planes) are estimated using the full covariance matrix. The cell e.s.d.'s are taken into account individually in the estimation of e.s.d.'s in distances, angles and torsion angles; correlations between e.s.d.'s in cell parameters are only used when they are defined by crystal symmetry. An approximate (isotropic) treatment of cell e.s.d.'s is used for estimating e.s.d.'s involving l.s. planes.
Refinement. Refinement of *F*^2^ against ALL reflections. The weighted *R*-factor *wR* and goodness of fit *S* are based on *F*^2^, conventional *R*-factors *R* are based on *F*, with *F* set to zero for negative *F*^2^. The threshold expression of *F*^2^ > σ(*F*^2^) is used only for calculating *R*-factors(gt) *etc*. and is not relevant to the choice of reflections for refinement. *R*-factors based on *F*^2^ are statistically about twice as large as those based on *F*, and *R*- factors based on ALL data will be even larger.

### Fractional atomic coordinates and isotropic or equivalent isotropic displacement parameters (Å^2^)

**Table:**

	*x*	*y*	*z*	*U*_iso_*/*U*_eq_	
Cl1A	0.65750 (6)	0.27225 (3)	0.07240 (3)	0.04616 (13)	
S1A	1.13306 (4)	1.04182 (3)	0.40859 (3)	0.02482 (8)	
F1A	1.42149 (12)	0.59713 (9)	0.08545 (7)	0.0464 (3)	
N1A	1.06028 (13)	0.73997 (8)	0.25101 (9)	0.0217 (2)	
N2A	1.09248 (13)	0.83652 (8)	0.29256 (9)	0.0229 (3)	
N3A	0.86452 (14)	0.97158 (9)	0.37237 (9)	0.0233 (3)	
N4A	0.37277 (16)	1.24996 (10)	0.53307 (12)	0.0402 (4)	
C1A	0.95272 (17)	0.52359 (11)	0.21546 (11)	0.0248 (3)	
H1AA	1.0519	0.5452	0.2463	0.030*	
C2A	0.88712 (18)	0.42599 (11)	0.17403 (11)	0.0284 (3)	
H2AA	0.9423	0.3823	0.1763	0.034*	
C3A	0.73902 (19)	0.39404 (11)	0.12930 (11)	0.0312 (4)	
C4A	0.65433 (18)	0.45760 (12)	0.12679 (12)	0.0324 (4)	
H4AA	0.5548	0.4354	0.0986	0.039*	
C5A	0.72013 (17)	0.55472 (12)	0.16696 (11)	0.0290 (3)	
H5AA	0.6640	0.5979	0.1648	0.035*	
C6A	0.87003 (16)	0.58924 (10)	0.21090 (10)	0.0229 (3)	
C7A	0.93212 (15)	0.69361 (10)	0.25151 (10)	0.0220 (3)	
C8A	0.88014 (16)	0.76421 (11)	0.29533 (11)	0.0242 (3)	
H8AA	0.7953	0.7563	0.3069	0.029*	
C9A	0.98191 (16)	0.85051 (10)	0.31872 (10)	0.0222 (3)	
C10A	0.97933 (16)	0.94805 (10)	0.36358 (10)	0.0225 (3)	
C11A	1.03671 (16)	1.11797 (11)	0.44212 (10)	0.0241 (3)	
H11A	1.0741	1.1836	0.4728	0.029*	
C12A	0.89653 (16)	1.06938 (10)	0.41774 (10)	0.0227 (3)	
C13A	0.78227 (16)	1.11019 (10)	0.43729 (10)	0.0231 (3)	
C14A	0.64515 (18)	1.05103 (12)	0.41109 (12)	0.0331 (4)	
H14B	0.6257	0.9860	0.3792	0.040*	
C15A	0.53762 (18)	1.08749 (12)	0.43178 (13)	0.0357 (4)	
H15B	0.4465	1.0473	0.4135	0.043*	
C16A	0.56653 (17)	1.18491 (11)	0.48025 (11)	0.0275 (3)	
C17A	0.70275 (18)	1.24542 (11)	0.50599 (11)	0.0283 (3)	
H17B	0.7219	1.3105	0.5376	0.034*	
C18A	0.80874 (18)	1.20810 (11)	0.48427 (11)	0.0276 (3)	
H18B	0.8990	1.2486	0.5011	0.033*	
C19A	0.45761 (17)	1.22203 (11)	0.50766 (12)	0.0311 (4)	
C20A	1.15274 (15)	0.70276 (10)	0.20723 (10)	0.0211 (3)	
C21A	1.29851 (17)	0.72700 (12)	0.25942 (11)	0.0282 (3)	
H21B	1.3348	0.7669	0.3218	0.034*	
C22A	1.39029 (18)	0.69147 (13)	0.21811 (12)	0.0333 (4)	
H22B	1.4884	0.7068	0.2521	0.040*	
C23A	1.33238 (18)	0.63320 (13)	0.12600 (12)	0.0310 (4)	
C24A	1.18802 (18)	0.60842 (12)	0.07246 (11)	0.0294 (3)	
H24B	1.1524	0.5684	0.0102	0.035*	
C25A	1.09718 (16)	0.64470 (11)	0.11382 (10)	0.0243 (3)	
H25B	0.9995	0.6302	0.0790	0.029*	
Cl1B	0.76527 (5)	−0.39437 (3)	−0.11384 (3)	0.04333 (12)	
S1B	1.14106 (4)	0.33994 (3)	0.36702 (3)	0.02914 (9)	
F1B	1.57725 (11)	−0.05590 (8)	0.10753 (9)	0.0484 (3)	
N1B	1.13119 (13)	0.06398 (8)	0.18871 (9)	0.0212 (2)	
N2B	1.14761 (13)	0.15432 (8)	0.24368 (9)	0.0224 (2)	
N3B	0.86803 (13)	0.25950 (8)	0.29038 (8)	0.0216 (2)	
N4B	0.31537 (16)	0.53818 (10)	0.35928 (12)	0.0417 (4)	
C1B	0.99178 (17)	−0.12120 (11)	0.01556 (10)	0.0251 (3)	
H1BA	1.0637	−0.0792	0.0097	0.030*	
C2B	0.93788 (18)	−0.21482 (11)	−0.04635 (11)	0.0286 (3)	
H2BA	0.9740	−0.2358	−0.0931	0.034*	
C3B	0.82978 (18)	−0.27638 (11)	−0.03761 (11)	0.0287 (3)	
C4B	0.77397 (17)	−0.24687 (11)	0.03145 (12)	0.0284 (3)	
H4BA	0.7011	−0.2890	0.0363	0.034*	
C5B	0.82882 (16)	−0.15338 (10)	0.09319 (11)	0.0246 (3)	
H5BA	0.7920	−0.1328	0.1397	0.029*	
C6B	0.93883 (16)	−0.08956 (10)	0.08655 (10)	0.0220 (3)	
C7B	0.99122 (16)	0.00952 (10)	0.15263 (10)	0.0212 (3)	
C8B	0.91412 (16)	0.06778 (10)	0.18710 (10)	0.0230 (3)	
H8BA	0.8164	0.0520	0.1760	0.028*	
C9B	1.01516 (16)	0.15581 (10)	0.24242 (10)	0.0219 (3)	
C10B	0.99350 (16)	0.24426 (10)	0.29498 (10)	0.0218 (3)	
C11B	1.02670 (17)	0.40573 (11)	0.39097 (11)	0.0267 (3)	
H11B	1.0558	0.4691	0.4303	0.032*	
C12B	0.88655 (16)	0.35308 (10)	0.34484 (10)	0.0220 (3)	
C13B	0.76062 (16)	0.38949 (10)	0.34638 (10)	0.0217 (3)	
C14B	0.62431 (17)	0.32800 (10)	0.31896 (11)	0.0255 (3)	
H14A	0.6121	0.2622	0.2988	0.031*	
C15B	0.50724 (17)	0.36374 (11)	0.32137 (12)	0.0289 (3)	
H15A	0.4171	0.3223	0.3030	0.035*	
C16B	0.52600 (17)	0.46296 (11)	0.35171 (11)	0.0258 (3)	
C17B	0.66100 (17)	0.52504 (10)	0.37804 (11)	0.0262 (3)	
H17A	0.6732	0.5908	0.3977	0.031*	
C18B	0.77587 (17)	0.48827 (10)	0.37473 (11)	0.0252 (3)	
H18A	0.8654	0.5298	0.3916	0.030*	
C19B	0.40724 (18)	0.50352 (11)	0.35543 (12)	0.0314 (4)	
C20B	1.25363 (15)	0.03680 (10)	0.17432 (10)	0.0216 (3)	
C21B	1.33796 (17)	0.09201 (11)	0.14291 (12)	0.0284 (3)	
H21A	1.3194	0.1487	0.1358	0.034*	
C22B	1.45039 (18)	0.06178 (12)	0.12222 (14)	0.0355 (4)	
H22A	1.5097	0.0982	0.1023	0.043*	
C23B	1.47150 (17)	−0.02352 (12)	0.13207 (13)	0.0321 (4)	
C24B	1.39172 (17)	−0.07789 (11)	0.16568 (12)	0.0303 (3)	
H24A	1.4112	−0.1342	0.1731	0.036*	
C25B	1.28151 (16)	−0.04656 (11)	0.18819 (11)	0.0258 (3)	
H25A	1.2270	−0.0810	0.2123	0.031*	

### Atomic displacement parameters (Å^2^)

**Table:**

	*U*^11^	*U*^22^	*U*^33^	*U*^12^	*U*^13^	*U*^23^
Cl1A	0.0592 (3)	0.0278 (2)	0.0379 (2)	−0.0061 (2)	0.0127 (2)	0.00634 (18)
S1A	0.02376 (18)	0.02641 (18)	0.02893 (19)	0.00997 (14)	0.01377 (15)	0.00987 (15)
F1A	0.0419 (6)	0.0755 (8)	0.0383 (6)	0.0401 (6)	0.0251 (5)	0.0185 (6)
N1A	0.0211 (6)	0.0218 (6)	0.0257 (6)	0.0092 (5)	0.0124 (5)	0.0070 (5)
N2A	0.0248 (6)	0.0218 (6)	0.0264 (6)	0.0097 (5)	0.0134 (5)	0.0082 (5)
N3A	0.0255 (6)	0.0244 (6)	0.0252 (6)	0.0113 (5)	0.0133 (5)	0.0092 (5)
N4A	0.0261 (7)	0.0277 (7)	0.0547 (10)	0.0079 (6)	0.0127 (7)	−0.0027 (7)
C1A	0.0226 (7)	0.0267 (7)	0.0274 (8)	0.0066 (6)	0.0109 (6)	0.0108 (6)
C2A	0.0348 (9)	0.0271 (7)	0.0290 (8)	0.0113 (7)	0.0162 (7)	0.0115 (6)
C3A	0.0383 (9)	0.0254 (7)	0.0253 (8)	−0.0012 (7)	0.0126 (7)	0.0063 (6)
C4A	0.0247 (8)	0.0376 (9)	0.0286 (8)	0.0005 (7)	0.0082 (7)	0.0080 (7)
C5A	0.0220 (7)	0.0341 (8)	0.0304 (8)	0.0084 (6)	0.0099 (6)	0.0093 (7)
C6A	0.0206 (7)	0.0264 (7)	0.0235 (7)	0.0067 (6)	0.0104 (6)	0.0082 (6)
C7A	0.0184 (7)	0.0269 (7)	0.0242 (7)	0.0089 (6)	0.0104 (6)	0.0096 (6)
C8A	0.0200 (7)	0.0291 (7)	0.0280 (8)	0.0102 (6)	0.0126 (6)	0.0100 (6)
C9A	0.0223 (7)	0.0258 (7)	0.0231 (7)	0.0114 (6)	0.0113 (6)	0.0092 (6)
C10A	0.0246 (7)	0.0249 (7)	0.0232 (7)	0.0105 (6)	0.0122 (6)	0.0099 (6)
C11A	0.0274 (8)	0.0240 (7)	0.0254 (7)	0.0104 (6)	0.0138 (6)	0.0086 (6)
C12A	0.0268 (7)	0.0253 (7)	0.0215 (7)	0.0125 (6)	0.0116 (6)	0.0097 (6)
C13A	0.0264 (7)	0.0261 (7)	0.0213 (7)	0.0132 (6)	0.0114 (6)	0.0083 (6)
C14A	0.0268 (8)	0.0263 (8)	0.0400 (9)	0.0114 (6)	0.0116 (7)	−0.0005 (7)
C15A	0.0231 (8)	0.0308 (8)	0.0427 (10)	0.0090 (7)	0.0099 (7)	−0.0020 (7)
C16A	0.0273 (8)	0.0290 (8)	0.0263 (8)	0.0155 (6)	0.0089 (6)	0.0058 (6)
C17A	0.0347 (9)	0.0233 (7)	0.0306 (8)	0.0135 (6)	0.0152 (7)	0.0079 (6)
C18A	0.0294 (8)	0.0246 (7)	0.0333 (8)	0.0096 (6)	0.0155 (7)	0.0106 (6)
C19A	0.0248 (8)	0.0251 (7)	0.0344 (9)	0.0091 (6)	0.0059 (7)	0.0001 (6)
C20A	0.0208 (7)	0.0236 (7)	0.0251 (7)	0.0104 (5)	0.0124 (6)	0.0104 (6)
C21A	0.0240 (8)	0.0363 (8)	0.0241 (8)	0.0106 (6)	0.0099 (6)	0.0071 (6)
C22A	0.0216 (8)	0.0509 (10)	0.0327 (9)	0.0183 (7)	0.0119 (7)	0.0153 (8)
C23A	0.0320 (9)	0.0443 (9)	0.0317 (9)	0.0253 (8)	0.0206 (7)	0.0170 (7)
C24A	0.0334 (9)	0.0349 (8)	0.0245 (8)	0.0169 (7)	0.0136 (7)	0.0092 (6)
C25A	0.0219 (7)	0.0289 (7)	0.0248 (7)	0.0108 (6)	0.0091 (6)	0.0100 (6)
Cl1B	0.0461 (3)	0.0250 (2)	0.0414 (2)	0.00970 (18)	0.0042 (2)	−0.00243 (17)
S1B	0.02187 (18)	0.02330 (18)	0.0385 (2)	0.00744 (14)	0.00972 (16)	0.00539 (16)
F1B	0.0310 (6)	0.0397 (6)	0.0831 (9)	0.0154 (5)	0.0356 (6)	0.0135 (6)
N1B	0.0209 (6)	0.0196 (5)	0.0251 (6)	0.0076 (5)	0.0106 (5)	0.0067 (5)
N2B	0.0247 (6)	0.0195 (6)	0.0252 (6)	0.0084 (5)	0.0119 (5)	0.0064 (5)
N3B	0.0237 (6)	0.0209 (6)	0.0221 (6)	0.0089 (5)	0.0104 (5)	0.0060 (5)
N4B	0.0287 (8)	0.0238 (7)	0.0653 (11)	0.0086 (6)	0.0160 (8)	0.0040 (7)
C1B	0.0272 (8)	0.0258 (7)	0.0251 (7)	0.0105 (6)	0.0100 (6)	0.0101 (6)
C2B	0.0352 (9)	0.0292 (8)	0.0226 (7)	0.0165 (7)	0.0093 (7)	0.0074 (6)
C3B	0.0327 (8)	0.0202 (7)	0.0257 (8)	0.0121 (6)	0.0019 (6)	0.0036 (6)
C4B	0.0230 (7)	0.0252 (7)	0.0340 (8)	0.0068 (6)	0.0064 (6)	0.0098 (6)
C5B	0.0216 (7)	0.0255 (7)	0.0268 (8)	0.0095 (6)	0.0085 (6)	0.0076 (6)
C6B	0.0211 (7)	0.0235 (7)	0.0215 (7)	0.0103 (6)	0.0059 (6)	0.0076 (5)
C7B	0.0215 (7)	0.0224 (7)	0.0223 (7)	0.0080 (5)	0.0097 (6)	0.0083 (5)
C8B	0.0210 (7)	0.0242 (7)	0.0256 (7)	0.0083 (6)	0.0104 (6)	0.0076 (6)
C9B	0.0239 (7)	0.0224 (7)	0.0231 (7)	0.0101 (6)	0.0107 (6)	0.0087 (6)
C10B	0.0241 (7)	0.0201 (6)	0.0232 (7)	0.0076 (5)	0.0099 (6)	0.0081 (5)
C11B	0.0267 (8)	0.0208 (7)	0.0306 (8)	0.0088 (6)	0.0102 (6)	0.0044 (6)
C12B	0.0252 (7)	0.0213 (7)	0.0221 (7)	0.0095 (6)	0.0105 (6)	0.0075 (5)
C13B	0.0247 (7)	0.0229 (7)	0.0188 (7)	0.0090 (6)	0.0095 (6)	0.0055 (5)
C14B	0.0271 (8)	0.0199 (7)	0.0281 (8)	0.0084 (6)	0.0105 (6)	0.0045 (6)
C15B	0.0251 (8)	0.0231 (7)	0.0364 (9)	0.0071 (6)	0.0120 (7)	0.0058 (6)
C16B	0.0251 (8)	0.0244 (7)	0.0280 (8)	0.0112 (6)	0.0109 (6)	0.0053 (6)
C17B	0.0304 (8)	0.0194 (7)	0.0280 (8)	0.0091 (6)	0.0124 (7)	0.0033 (6)
C18B	0.0250 (7)	0.0218 (7)	0.0269 (8)	0.0062 (6)	0.0105 (6)	0.0044 (6)
C19B	0.0275 (8)	0.0212 (7)	0.0408 (9)	0.0061 (6)	0.0122 (7)	0.0035 (7)
C20B	0.0183 (7)	0.0229 (7)	0.0244 (7)	0.0073 (5)	0.0093 (6)	0.0063 (6)
C21B	0.0261 (8)	0.0227 (7)	0.0408 (9)	0.0075 (6)	0.0168 (7)	0.0119 (6)
C22B	0.0294 (9)	0.0306 (8)	0.0555 (11)	0.0076 (7)	0.0270 (8)	0.0154 (8)
C23B	0.0203 (7)	0.0299 (8)	0.0472 (10)	0.0095 (6)	0.0176 (7)	0.0067 (7)
C24B	0.0258 (8)	0.0259 (7)	0.0416 (9)	0.0126 (6)	0.0128 (7)	0.0111 (7)
C25B	0.0236 (7)	0.0268 (7)	0.0321 (8)	0.0100 (6)	0.0132 (6)	0.0126 (6)

### Geometric parameters (Å, °)

**Table:**

Cl1A—C3A	1.7401 (16)	Cl1B—C3B	1.7428 (15)
S1A—C11A	1.7044 (15)	S1B—C11B	1.7055 (15)
S1A—C10A	1.7325 (16)	S1B—C10B	1.7312 (15)
F1A—C23A	1.3617 (17)	F1B—C23B	1.3647 (17)
N1A—N2A	1.3622 (16)	N1B—N2B	1.3570 (16)
N1A—C7A	1.3761 (18)	N1B—C7B	1.3749 (18)
N1A—C20A	1.4273 (17)	N1B—C20B	1.4314 (18)
N2A—C9A	1.3377 (18)	N2B—C9B	1.3411 (18)
N3A—C10A	1.3063 (18)	N3B—C10B	1.3124 (19)
N3A—C12A	1.3879 (18)	N3B—C12B	1.3895 (18)
N4A—C19A	1.148 (2)	N4B—C19B	1.148 (2)
C1A—C2A	1.390 (2)	C1B—C2B	1.390 (2)
C1A—C6A	1.397 (2)	C1B—C6B	1.398 (2)
C1A—H1AA	0.9300	C1B—H1BA	0.9300
C2A—C3A	1.386 (2)	C2B—C3B	1.384 (2)
C2A—H2AA	0.9300	C2B—H2BA	0.9300
C3A—C4A	1.385 (2)	C3B—C4B	1.387 (2)
C4A—C5A	1.384 (2)	C4B—C5B	1.388 (2)
C4A—H4AA	0.9300	C4B—H4BA	0.9300
C5A—C6A	1.403 (2)	C5B—C6B	1.400 (2)
C5A—H5AA	0.9300	C5B—H5BA	0.9300
C6A—C7A	1.473 (2)	C6B—C7B	1.474 (2)
C7A—C8A	1.379 (2)	C7B—C8B	1.3808 (19)
C8A—C9A	1.402 (2)	C8B—C9B	1.402 (2)
C8A—H8AA	0.9300	C8B—H8BA	0.9300
C9A—C10A	1.459 (2)	C9B—C10B	1.459 (2)
C11A—C12A	1.372 (2)	C11B—C12B	1.369 (2)
C11A—H11A	0.9300	C11B—H11B	0.9300
C12A—C13A	1.471 (2)	C12B—C13B	1.473 (2)
C13A—C14A	1.396 (2)	C13B—C14B	1.400 (2)
C13A—C18A	1.401 (2)	C13B—C18B	1.401 (2)
C14A—C15A	1.384 (2)	C14B—C15B	1.387 (2)
C14A—H14B	0.9300	C14B—H14A	0.9300
C15A—C16A	1.396 (2)	C15B—C16B	1.402 (2)
C15A—H15B	0.9300	C15B—H15A	0.9300
C16A—C17A	1.398 (2)	C16B—C17B	1.397 (2)
C16A—C19A	1.444 (2)	C16B—C19B	1.445 (2)
C17A—C18A	1.381 (2)	C17B—C18B	1.377 (2)
C17A—H17B	0.9300	C17B—H17A	0.9300
C18A—H18B	0.9300	C18B—H18A	0.9300
C20A—C25A	1.385 (2)	C20B—C25B	1.388 (2)
C20A—C21A	1.385 (2)	C20B—C21B	1.388 (2)
C21A—C22A	1.389 (2)	C21B—C22B	1.390 (2)
C21A—H21B	0.9300	C21B—H21A	0.9300
C22A—C23A	1.370 (2)	C22B—C23B	1.376 (2)
C22A—H22B	0.9300	C22B—H22A	0.9300
C23A—C24A	1.377 (2)	C23B—C24B	1.377 (2)
C24A—C25A	1.386 (2)	C24B—C25B	1.387 (2)
C24A—H24B	0.9300	C24B—H24A	0.9300
C25A—H25B	0.9300	C25B—H25A	0.9300
			
C11A—S1A—C10A	89.00 (7)	C11B—S1B—C10B	88.81 (7)
N2A—N1A—C7A	112.26 (11)	N2B—N1B—C7B	112.35 (11)
N2A—N1A—C20A	117.81 (11)	N2B—N1B—C20B	119.34 (12)
C7A—N1A—C20A	129.66 (12)	C7B—N1B—C20B	128.31 (12)
C9A—N2A—N1A	104.36 (12)	C9B—N2B—N1B	104.17 (12)
C10A—N3A—C12A	110.04 (13)	C10B—N3B—C12B	109.82 (12)
C2A—C1A—C6A	120.19 (15)	C2B—C1B—C6B	120.61 (14)
C2A—C1A—H1AA	119.9	C2B—C1B—H1BA	119.7
C6A—C1A—H1AA	119.9	C6B—C1B—H1BA	119.7
C3A—C2A—C1A	119.69 (15)	C3B—C2B—C1B	119.22 (15)
C3A—C2A—H2AA	120.2	C3B—C2B—H2BA	120.4
C1A—C2A—H2AA	120.2	C1B—C2B—H2BA	120.4
C4A—C3A—C2A	121.18 (15)	C2B—C3B—C4B	121.55 (14)
C4A—C3A—Cl1A	119.05 (13)	C2B—C3B—Cl1B	119.01 (13)
C2A—C3A—Cl1A	119.75 (14)	C4B—C3B—Cl1B	119.42 (13)
C5A—C4A—C3A	118.95 (15)	C3B—C4B—C5B	118.84 (15)
C5A—C4A—H4AA	120.5	C3B—C4B—H4BA	120.6
C3A—C4A—H4AA	120.5	C5B—C4B—H4BA	120.6
C4A—C5A—C6A	121.13 (15)	C4B—C5B—C6B	120.98 (14)
C4A—C5A—H5AA	119.4	C4B—C5B—H5BA	119.5
C6A—C5A—H5AA	119.4	C6B—C5B—H5BA	119.5
C1A—C6A—C5A	118.80 (14)	C1B—C6B—C5B	118.80 (13)
C1A—C6A—C7A	123.26 (13)	C1B—C6B—C7B	122.35 (13)
C5A—C6A—C7A	117.93 (14)	C5B—C6B—C7B	118.81 (13)
N1A—C7A—C8A	105.87 (13)	N1B—C7B—C8B	106.21 (12)
N1A—C7A—C6A	124.12 (13)	N1B—C7B—C6B	124.85 (13)
C8A—C7A—C6A	130.00 (13)	C8B—C7B—C6B	128.87 (13)
C7A—C8A—C9A	105.62 (13)	C7B—C8B—C9B	105.04 (13)
C7A—C8A—H8AA	127.2	C7B—C8B—H8BA	127.5
C9A—C8A—H8AA	127.2	C9B—C8B—H8BA	127.5
N2A—C9A—C8A	111.89 (13)	N2B—C9B—C8B	112.23 (13)
N2A—C9A—C10A	118.99 (13)	N2B—C9B—C10B	118.76 (13)
C8A—C9A—C10A	129.10 (13)	C8B—C9B—C10B	129.00 (14)
N3A—C10A—C9A	123.79 (14)	N3B—C10B—C9B	125.06 (13)
N3A—C10A—S1A	115.43 (11)	N3B—C10B—S1B	115.54 (11)
C9A—C10A—S1A	120.78 (11)	C9B—C10B—S1B	119.37 (11)
C12A—C11A—S1A	110.82 (11)	C12B—C11B—S1B	111.18 (11)
C12A—C11A—H11A	124.6	C12B—C11B—H11B	124.4
S1A—C11A—H11A	124.6	S1B—C11B—H11B	124.4
C11A—C12A—N3A	114.71 (13)	C11B—C12B—N3B	114.62 (13)
C11A—C12A—C13A	126.63 (14)	C11B—C12B—C13B	125.19 (13)
N3A—C12A—C13A	118.64 (13)	N3B—C12B—C13B	120.14 (13)
C14A—C13A—C18A	118.52 (14)	C14B—C13B—C18B	118.50 (13)
C14A—C13A—C12A	120.22 (13)	C14B—C13B—C12B	121.88 (13)
C18A—C13A—C12A	121.24 (14)	C18B—C13B—C12B	119.61 (13)
C15A—C14A—C13A	121.12 (15)	C15B—C14B—C13B	120.98 (14)
C15A—C14A—H14B	119.4	C15B—C14B—H14A	119.5
C13A—C14A—H14B	119.4	C13B—C14B—H14A	119.5
C14A—C15A—C16A	119.65 (16)	C14B—C15B—C16B	119.36 (14)
C14A—C15A—H15B	120.2	C14B—C15B—H15A	120.3
C16A—C15A—H15B	120.2	C16B—C15B—H15A	120.3
C15A—C16A—C17A	119.99 (14)	C17B—C16B—C15B	120.22 (14)
C15A—C16A—C19A	119.77 (15)	C17B—C16B—C19B	118.43 (13)
C17A—C16A—C19A	120.17 (14)	C15B—C16B—C19B	121.34 (14)
C18A—C17A—C16A	119.70 (14)	C18B—C17B—C16B	119.62 (14)
C18A—C17A—H17B	120.2	C18B—C17B—H17A	120.2
C16A—C17A—H17B	120.2	C16B—C17B—H17A	120.2
C17A—C18A—C13A	121.01 (15)	C17B—C18B—C13B	121.30 (14)
C17A—C18A—H18B	119.5	C17B—C18B—H18A	119.3
C13A—C18A—H18B	119.5	C13B—C18B—H18A	119.3
N4A—C19A—C16A	176.7 (2)	N4B—C19B—C16B	178.14 (17)
C25A—C20A—C21A	120.93 (14)	C25B—C20B—C21B	121.52 (13)
C25A—C20A—N1A	120.26 (13)	C25B—C20B—N1B	119.10 (13)
C21A—C20A—N1A	118.80 (13)	C21B—C20B—N1B	119.32 (13)
C20A—C21A—C22A	119.68 (15)	C20B—C21B—C22B	119.32 (14)
C20A—C21A—H21B	120.2	C20B—C21B—H21A	120.3
C22A—C21A—H21B	120.2	C22B—C21B—H21A	120.3
C23A—C22A—C21A	118.22 (15)	C23B—C22B—C21B	118.09 (15)
C23A—C22A—H22B	120.9	C23B—C22B—H22A	121.0
C21A—C22A—H22B	120.9	C21B—C22B—H22A	121.0
F1A—C23A—C22A	118.52 (15)	F1B—C23B—C22B	118.39 (15)
F1A—C23A—C24A	118.24 (15)	F1B—C23B—C24B	118.19 (14)
C22A—C23A—C24A	123.24 (15)	C22B—C23B—C24B	123.42 (14)
C23A—C24A—C25A	118.27 (15)	C23B—C24B—C25B	118.34 (14)
C23A—C24A—H24B	120.9	C23B—C24B—H24A	120.8
C25A—C24A—H24B	120.9	C25B—C24B—H24A	120.8
C20A—C25A—C24A	119.63 (14)	C24B—C25B—C20B	119.20 (14)
C20A—C25A—H25B	120.2	C24B—C25B—H25A	120.4
C24A—C25A—H25B	120.2	C20B—C25B—H25A	120.4
			
C7A—N1A—N2A—C9A	−0.46 (16)	C7B—N1B—N2B—C9B	0.54 (16)
C20A—N1A—N2A—C9A	174.20 (12)	C20B—N1B—N2B—C9B	−179.95 (12)
C6A—C1A—C2A—C3A	−0.9 (2)	C6B—C1B—C2B—C3B	0.7 (2)
C1A—C2A—C3A—C4A	−1.4 (2)	C1B—C2B—C3B—C4B	0.0 (2)
C1A—C2A—C3A—Cl1A	176.82 (12)	C1B—C2B—C3B—Cl1B	−178.65 (12)
C2A—C3A—C4A—C5A	2.3 (2)	C2B—C3B—C4B—C5B	−0.3 (2)
Cl1A—C3A—C4A—C5A	−175.97 (12)	Cl1B—C3B—C4B—C5B	178.40 (12)
C3A—C4A—C5A—C6A	−0.9 (2)	C3B—C4B—C5B—C6B	−0.2 (2)
C2A—C1A—C6A—C5A	2.3 (2)	C2B—C1B—C6B—C5B	−1.1 (2)
C2A—C1A—C6A—C7A	−178.81 (14)	C2B—C1B—C6B—C7B	−178.93 (14)
C4A—C5A—C6A—C1A	−1.4 (2)	C4B—C5B—C6B—C1B	0.8 (2)
C4A—C5A—C6A—C7A	179.62 (14)	C4B—C5B—C6B—C7B	178.76 (14)
N2A—N1A—C7A—C8A	0.16 (17)	N2B—N1B—C7B—C8B	−0.64 (16)
C20A—N1A—C7A—C8A	−173.70 (14)	C20B—N1B—C7B—C8B	179.92 (13)
N2A—N1A—C7A—C6A	179.59 (13)	N2B—N1B—C7B—C6B	176.52 (13)
C20A—N1A—C7A—C6A	5.7 (2)	C20B—N1B—C7B—C6B	−2.9 (2)
C1A—C6A—C7A—N1A	37.1 (2)	C1B—C6B—C7B—N1B	−36.7 (2)
C5A—C6A—C7A—N1A	−143.99 (15)	C5B—C6B—C7B—N1B	145.41 (14)
C1A—C6A—C7A—C8A	−143.60 (17)	C1B—C6B—C7B—C8B	139.76 (16)
C5A—C6A—C7A—C8A	35.3 (2)	C5B—C6B—C7B—C8B	−38.1 (2)
N1A—C7A—C8A—C9A	0.20 (16)	N1B—C7B—C8B—C9B	0.44 (16)
C6A—C7A—C8A—C9A	−179.19 (15)	C6B—C7B—C8B—C9B	−176.56 (14)
N1A—N2A—C9A—C8A	0.59 (17)	N1B—N2B—C9B—C8B	−0.24 (16)
N1A—N2A—C9A—C10A	−178.03 (12)	N1B—N2B—C9B—C10B	−179.58 (12)
C7A—C8A—C9A—N2A	−0.51 (18)	C7B—C8B—C9B—N2B	−0.13 (17)
C7A—C8A—C9A—C10A	177.94 (15)	C7B—C8B—C9B—C10B	179.12 (14)
C12A—N3A—C10A—C9A	−179.85 (13)	C12B—N3B—C10B—C9B	−176.26 (13)
C12A—N3A—C10A—S1A	−0.36 (16)	C12B—N3B—C10B—S1B	1.59 (16)
N2A—C9A—C10A—N3A	163.76 (14)	N2B—C9B—C10B—N3B	170.87 (14)
C8A—C9A—C10A—N3A	−14.6 (3)	C8B—C9B—C10B—N3B	−8.3 (2)
N2A—C9A—C10A—S1A	−15.70 (19)	N2B—C9B—C10B—S1B	−6.90 (18)
C8A—C9A—C10A—S1A	165.95 (13)	C8B—C9B—C10B—S1B	173.89 (12)
C11A—S1A—C10A—N3A	0.31 (12)	C11B—S1B—C10B—N3B	−1.16 (12)
C11A—S1A—C10A—C9A	179.81 (13)	C11B—S1B—C10B—C9B	176.82 (12)
C10A—S1A—C11A—C12A	−0.15 (12)	C10B—S1B—C11B—C12B	0.34 (12)
S1A—C11A—C12A—N3A	−0.01 (17)	S1B—C11B—C12B—N3B	0.48 (17)
S1A—C11A—C12A—C13A	178.17 (12)	S1B—C11B—C12B—C13B	−176.84 (11)
C10A—N3A—C12A—C11A	0.24 (18)	C10B—N3B—C12B—C11B	−1.32 (18)
C10A—N3A—C12A—C13A	−178.10 (13)	C10B—N3B—C12B—C13B	176.15 (12)
C11A—C12A—C13A—C14A	−177.63 (16)	C11B—C12B—C13B—C14B	−163.03 (15)
N3A—C12A—C13A—C14A	0.5 (2)	N3B—C12B—C13B—C14B	19.8 (2)
C11A—C12A—C13A—C18A	0.8 (2)	C11B—C12B—C13B—C18B	17.9 (2)
N3A—C12A—C13A—C18A	178.91 (14)	N3B—C12B—C13B—C18B	−159.31 (13)
C18A—C13A—C14A—C15A	−0.8 (3)	C18B—C13B—C14B—C15B	−1.2 (2)
C12A—C13A—C14A—C15A	177.70 (16)	C12B—C13B—C14B—C15B	179.68 (14)
C13A—C14A—C15A—C16A	−0.5 (3)	C13B—C14B—C15B—C16B	0.0 (2)
C14A—C15A—C16A—C17A	1.3 (3)	C14B—C15B—C16B—C17B	0.8 (2)
C14A—C15A—C16A—C19A	−175.72 (17)	C14B—C15B—C16B—C19B	−179.99 (15)
C15A—C16A—C17A—C18A	−0.8 (2)	C15B—C16B—C17B—C18B	−0.5 (2)
C19A—C16A—C17A—C18A	176.16 (15)	C19B—C16B—C17B—C18B	−179.70 (15)
C16A—C17A—C18A—C13A	−0.4 (2)	C16B—C17B—C18B—C13B	−0.7 (2)
C14A—C13A—C18A—C17A	1.2 (2)	C14B—C13B—C18B—C17B	1.6 (2)
C12A—C13A—C18A—C17A	−177.22 (14)	C12B—C13B—C18B—C17B	−179.31 (14)
N2A—N1A—C20A—C25A	−124.58 (15)	N2B—N1B—C20B—C25B	129.87 (15)
C7A—N1A—C20A—C25A	49.0 (2)	C7B—N1B—C20B—C25B	−50.7 (2)
N2A—N1A—C20A—C21A	54.44 (18)	N2B—N1B—C20B—C21B	−52.93 (19)
C7A—N1A—C20A—C21A	−131.98 (16)	C7B—N1B—C20B—C21B	126.48 (16)
C25A—C20A—C21A—C22A	−1.0 (2)	C25B—C20B—C21B—C22B	1.9 (3)
N1A—C20A—C21A—C22A	179.96 (14)	N1B—C20B—C21B—C22B	−175.22 (15)
C20A—C21A—C22A—C23A	0.2 (3)	C20B—C21B—C22B—C23B	1.3 (3)
C21A—C22A—C23A—F1A	−179.16 (15)	C21B—C22B—C23B—F1B	176.88 (16)
C21A—C22A—C23A—C24A	0.2 (3)	C21B—C22B—C23B—C24B	−3.3 (3)
F1A—C23A—C24A—C25A	179.68 (14)	F1B—C23B—C24B—C25B	−178.21 (15)
C22A—C23A—C24A—C25A	0.3 (3)	C22B—C23B—C24B—C25B	2.0 (3)
C21A—C20A—C25A—C24A	1.6 (2)	C23B—C24B—C25B—C20B	1.3 (2)
N1A—C20A—C25A—C24A	−179.45 (13)	C21B—C20B—C25B—C24B	−3.3 (2)
C23A—C24A—C25A—C20A	−1.2 (2)	N1B—C20B—C25B—C24B	173.87 (14)

### Hydrogen-bond geometry (Å, °)

**Table:**

Cg1 and Cg2 are the centroids of the C1A–C6A and C1B–C6B rings, respectively.

**Table:**

*D*—H···*A*	*D*—H	H···*A*	*D*···*A*	*D*—H···*A*
C5A—H5AA···F1A^i^	0.93	2.39	3.149 (2)	138
C8B—H8BA···F1B^i^	0.93	2.42	3.283 (2)	154
C17B—H17A···N4A^ii^	0.93	2.54	3.419 (2)	159
C17A—H17B···N4B^ii^	0.93	2.58	3.453 (2)	156
C25B—H25A···N2A^iii^	0.93	2.53	3.457 (2)	175
C24A—H24B···Cg1^iv^	0.93	2.96	3.7811 (18)	148
C21B—H21A···Cg2^v^	0.93	2.97	3.6423 (19)	131

Symmetry codes: (i) *x*−1, *y*, *z*; (ii) −*x*+1, −*y*+2, −*z*+1; (iii) *x*, *y*−1, *z*; (iv) −*x*+2, −*y*+1, −*z*; (v) −*x*+2, −*y*, −*z*.
